# Causal association between particulate matter 2.5 and Alzheimer’s disease: a Mendelian randomization study

**DOI:** 10.3389/fpubh.2024.1343915

**Published:** 2024-05-30

**Authors:** Meijuan Dang, Ye Li, Lili Zhao, Tao Li, Ziwei Lu, Jialiang Lu, Yuxuan Feng, Yang Yang, Fangcun Li, Fan Tang, Xiaoya Wang, Yating Jian, Heying Wang, Lei Zhang, Hong Fan, Guilian Zhang

**Affiliations:** Department of Neurology, The Second Affiliated Hospital of Xi'an Jiaotong University, Xi'an, China

**Keywords:** Alzheimer’s disease, particulate matter, Mendelian randomization, neurodegenerative diseases, air pollution

## Abstract

**Background:**

Although epidemiological evidence implies a link between exposure to particulate matter (PM) and Alzheimer’s disease (AD), establishing causality remains a complex endeavor. In the present study, we used Mendelian randomization (MR) as a robust analytical approach to explore the potential causal relationship between PM exposure and AD risk. We also explored the potential associations between PM exposure and other neurodegenerative diseases.

**Methods:**

Drawing on extensive genome-wide association studies related to PM exposure, we identified the instrumental variables linked to individual susceptibility to PM. Using summary statistics from five distinct neurodegenerative diseases, we conducted two-sample MR analyses to gauge the causal impact of PM on the risk of developing these diseases. Sensitivity analyses were undertaken to evaluate the robustness of our findings. Additionally, we executed multivariable MR (MVMR) to validate the significant causal associations identified in the two-sample MR analyses, by adjusting for potential confounding risk factors.

**Results:**

Our MR analysis identified a notable association between genetically predicted PM2.5 (PM with a diameter of 2.5 μm or less) exposure and an elevated risk of AD (odds ratio, 2.160; 95% confidence interval, 1.481 to 3.149; *p* < 0.001). A sensitivity analysis supported the robustness of the observed association, thus alleviating concerns related to pleiotropy. No discernible causal relationship was identified between PM and any other neurodegenerative diseases. MVMR analyses—adjusting for smoking, alcohol use, education, stroke, hearing loss, depression, and hypertension—confirmed a persistent causal relationship between PM2.5 and AD. Sensitivity analyses, including MR-Egger and weighted median analyses, also supported this causal association.

**Conclusion:**

The present MR study provides evidence to support a plausible causal connection between PM2.5 exposure and AD. The results emphasize the importance of contemplating air quality interventions as a public health strategy for reducing AD risk.

## 1 Introduction

Alzheimer’s disease (AD) is among the most prevalent neurodegenerative conditions; it manifests as memory loss, progressive cognitive decline, and compromised daily functioning. The World Alzheimer Report 2023 indicated that there were 55 million people with dementia worldwide in 2019, and the authors projected that this number will escalate to 139 million by 2050 (1). In an aging global population, it is imperative to investigate the causative factors of neurodegenerative diseases such as AD for the collective benefit of society. Characterized by its multifaceted nature, a precise understanding of the specific pathogenesis of AD remains lacking. Over the past decades, researchers have identified numerous risk factors for AD, including lower educational attainment, smoking, and hypertension (2). Intriguingly, emerging evidence suggests a potential association between air pollution and AD development (3).

Air pollution is a pervasive global threat to both human health and the environment. Although our understanding of the health implications of air pollution has progressed, the effects of this pollution on the central nervous system remain only partially elucidated. Notably, we have limited knowledge regarding the impact of air pollution on neurodegenerative diseases (4). However, epidemiological evidence suggests an increased prevalence and incidence of AD with exposure to air pollution (5), and particularly to particulate matter (PM), which can cause profound transcriptional dysregulation and accelerate AD-related pathology (6). Among the various sizes of PM, PM2.5 (PM with a diameter of 2.5 μm or less) has attracted considerable attention because it can penetrate deep into the respiratory system. Moreover, connections have been reported between PM and neurodegenerative diseases other than AD, including amyotrophic lateral sclerosis (ALS), dementia with Lewy bodies (DLB), multiple sclerosis (MS), and Parkinson’s disease (PD) (7). Nevertheless, it is difficult to establish a definitive causal link between PM and neurodegenerative diseases because of the susceptibility of such associations to confounding factors.

Given the inherent confounding that is present in observational studies, we opted to take a Mendelian randomization (MR) approach to investigate the relationship between PM and neurodegenerative diseases. MR, a genetic epidemiology method, uses instrumental variables (IVs) that represent one or more exposures (typically single nucleotide polymorphisms [SNPs]) as unbiased proxies to assess the causal association of these exposures with disease (8). The random assignment and fixation of genetic variants at conception allow the MR method to mitigate the impact of confounding factors. Additionally, because genetic variants are predetermined before disease onset, bias stemming from reverse causation—a challenge inherent in observational studies—can be substantially reduced. MR is therefore regarded as an acceptable approach for investigating causal relationships, and is the second-most robust method after randomized controlled trials.

To date, no MR studies have comprehensively explored the causal relationships between PM and neurodegenerative diseases. In the present study, we therefore used a two-sample MR method to estimate the causal effects of PM on neurodegenerative diseases (namely, AD, ALS, DLB, PD, and MS). Moreover, we used multivariable MR analysis (MVMR) to assess the causal effects while adjusting for potential confounding factors. Our results may offer insights into the potential role of PM in the prevention and treatment of neurodegenerative diseases.

## 2 Materials and methods

### 2.1 Study design

**Figure 1:**
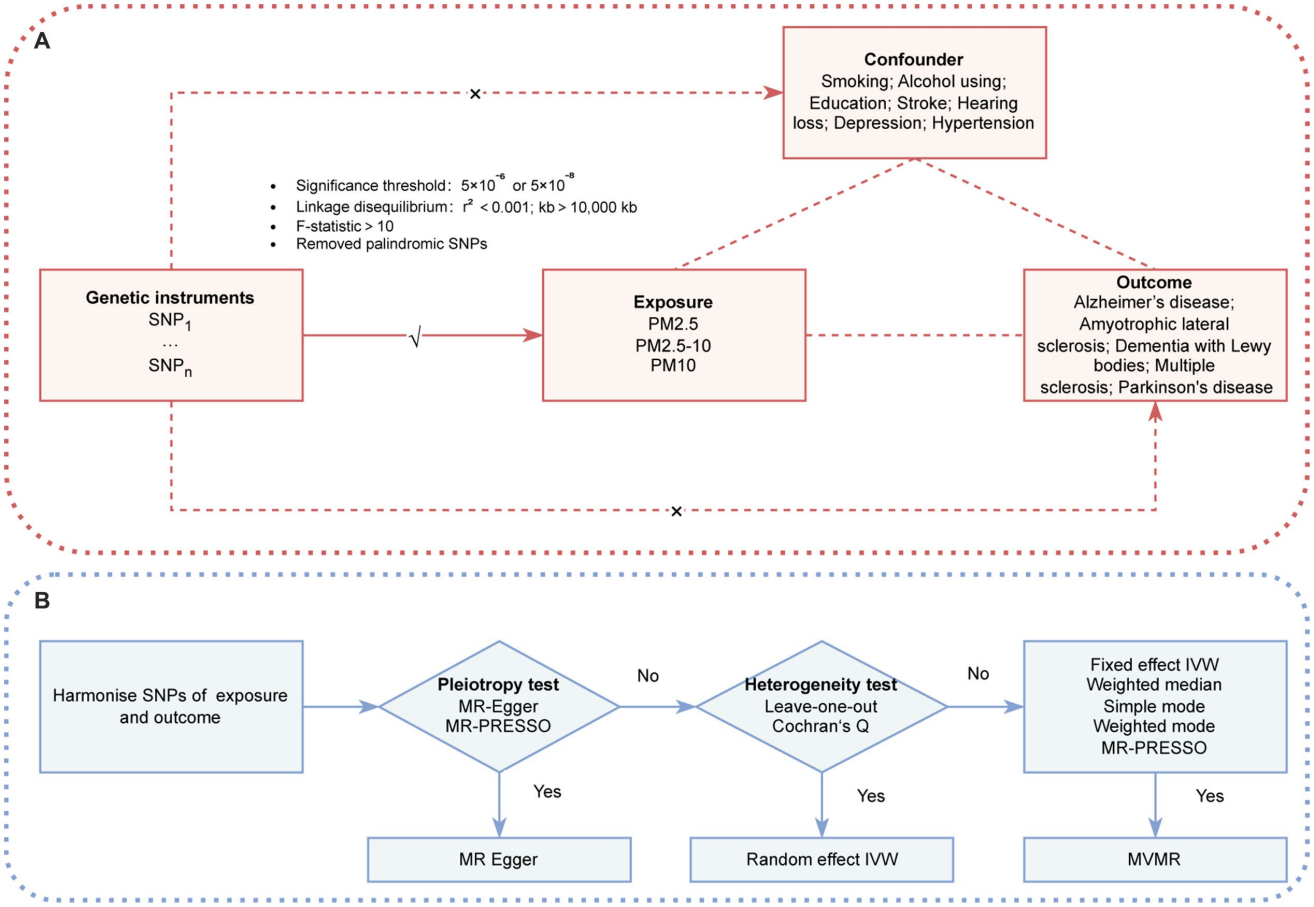
**(A)** Three assumption of MR. **(B)** The conceptual schematic of the MR research design. IVW, inverse variance weighted; MR-PRESSO, MR pleiotropy residual sum and outlier; PM, particulate matter; SNP, single nucleotide polymorphism.

The MR analysis in this study adhered to the following criteria (Figure 1A): (1) the IVs exhibited a robust association with the exposures; (2) the IVs were independent of potential confounders; and (3) the IVs affected the outcome via the exposure of interest only, and not via any other alternative pathways. Our findings are reported in accordance with the Strengthening the Reporting of Observational Studies in Epidemiology-MR guidelines (9). The two-sample MR analysis was conducted to identify any potential causal associations between PM (PM2.5, PM2.5–10 [PM with a diameter of 2.5–10 μm], and PM10 [PM with a diameter of a diameter of 10 μm or less]) and neurodegenerative diseases (AD, ALS, DLB, PD, and MS). To ensure the robustness of our conclusions, a MVMR was conducted to adjust for potential risk factors. Because there was no direct interaction with human participants in this research, informed consent was deemed unnecessary.

### 2.2 Genome-wide association study (GWAS) summary data for PM

The data sources are detailed in Table 1. Genetic variants associated with PM2.5, PM2.5–10, and PM10 were extracted from a published GWAS that involved 423,796 individuals of European descent from the UK Biobank (10). The present study built upon the European Study of Cohorts for Air Pollution Effects, and used the Land Use Regression model to estimate concentrations of PM pollution at the residential addresses of participants.

**Table 1:** Characteristics of summary genome-wide association studies.

Trait	Year	PMID	Sample size	Number of SNPs	Population	GWAS ID
PM						
PM2.5	2018	22963366	423,796	9,851,867	European	ukb-b-10817
PM2.5–10	2018	22963366	423,796	9,851,867	European	ukb-b-12963
PM10	2018	22963366	423,796	9,851,867	European	ukb-b-589
Neurodegenerative disease						
AD	2019	30820047	63,926	10,528,610	European	ieu-b-2
ALS	2021	34873335	138,086	10,427,126	European	ebi-a-GCST90027164
DLB	2021	33589841	6,618	7,593,175	European	ebi-a-GCST90001390
PD	2019	31701892	482,730	17,891,936	European	ieu-b-7
MS	2019	31604244	115,803	6,304,359	European	ieu-b-18
Confounder						
Smoking	2019	30643251	341,427	11,894,779	European	ieu-b-24
Alcoholic drinks	2019	30643251	335,394	11,887,865	European	ieu-b-73
Years of schooling	2022	35534559	54,986	7,210,035	European	ieu-b-4835
Stroke	2018	29531354	446,696	8,211,693	European	ebi-a-GCST006906
Essential hypertension	2021	33959723	484,598	9,587,836	European	ebi-a-GCST90038608
Depression	2021	34594039	449,414	24,184,163	European	ebi-a-GCST90018833
Hearing loss	2021	34594039	489,493	24,181,241	European	ebi-a-GCST90018857

SNPs, single nucleotide polymorphisms; GWAS, Genome-Wide Association Study; AD, Alzheimer’s disease; ALS, amyotrophic lateral sclerosis; DLB, dementia with Lewy bodies; PD, Parkinson’s disease; MS, multiple sclerosis.

### 2.3 GWAS summary data for neurodegenerative diseases

For AD, summary-level data were obtained from a GWAS meta-analysis conducted by the International Genomics of Alzheimer’s Project, which encompassed 21,982 AD cases and 41,944 controls (11). The ALS summary statistics were sourced from a recent GWAS meta-analysis involving 138,086 participants (27,205 cases and 110,881 controls) (12). The dataset for DLB comprised 2,981 patients diagnosed with DLB and 4,391 healthy controls, recruited by 44 institutions or consortia (13). The PD GWAS meta-analysis used summary statistics from 33,674 European PD patients and 449,056 controls, and was conducted by the International PD Genomics Consortium (14). The MS data were obtained from the International MS Genetics Consortium, and included 47,429 MS cases and 68,374 controls (15).

### 2.4 GWAS summary data for confounders

We considered several factors as potential confounders of the exposure–outcome relationship. Smoking and alcohol consumption data were retrieved from the GWAS and Sequencing Consortium of Alcohol and Nicotine Use (16). The summary statistics for years of education involved 54,986 European individuals (17). For genetic associations with stroke, publicly available summarized data from the MEGASTROKE consortium were used, comprising 446,696 individuals of European ancestry (406,111 controls and 40,585 cases of any stroke) (18). Summary statistics for hypertension were obtained from a GWAS of 484,598 individuals of European descent (19). GWAS depression data were obtained from a published GWAS of 13,559 patients with depression and 435,855 healthy controls of European ancestry (20). Additionally, summary statistics for hearing loss were retrieved from a GWAS of 14,654 cases and 474,839 controls of European descent (20).

### 2.5 IV selection

To ensure the credibility and precision of our conclusions regarding the causal relationship between PM and neurodegenerative diseases, we used the following rigorous criteria to select valid IVs. (1) The association between SNPs and phenotypes reached the locus-wide significance threshold (*p* < 5 × 10^−8^) for the selection of potential IVs. Unfortunately, only a limited number of SNPs met these criteria for PM2.5, PM2.5–10, and confounders. To explore additional relationships between PM and neurodegenerative diseases and to achieve a more comprehensive analysis, a threshold of *p* < 5 × 10^−6^ was applied to identify SNPs, except for PM10. (2) To ensure the independence of each SNP, a clumping procedure was implemented; only SNPs with the lowest *p*-values were retained. The linkage disequilibrium threshold for clumping was set at r^2^ < 0.001, with a clumping window width of 10,000 kb. (3) To assess the strength of IVs, the F-statistic was calculated as follows: R^2^ × (N − 2) / (1 − R^2^), where N is the sample size and R^2^ is the proportion of the variance of the trait that is accounted for by the SNP. The R^2^ value was calculated using the following formula: R^2^ = (2 × EAF × (1 − EAF) × Beta^2^) / [(2 × EAF × (1 − EAF) × Beta^2^) + (2 × EAF × (1 − EAF) × N × SE^2^)]. The F-statistics for all SNPs were greater than 10, indicating that the weak instrument bias was negligible (21). (4) Palindromic SNPs were excluded from the analysis to ensure that the effects of SNPs on exposures corresponded to the same allele as the effects on outcomes. Specific SNP information, along with matching R^2^ and F-statistic values, is provided in Supplementary Table S1. Furthermore, Supplementary Table S2 provide the SNP filtering process.

### 2.6 Statistical analysis

All statistical analyses were conducted using R version 4.3.0 (R Foundation for Statistical Computing, Austria) with the “TwoSampleMR,” “MendelianRandomization,” and “MR PRESSO” R packages. Bonferroni correction was used to identify false-positive results caused by multiple testing. A stringent multiple test significance threshold was established at 0.0003 (0.05 / 15). Significance values falling within 0.0003 and 0.05 were deemed suggestively significant. This approach ensured a rigorous control for Type I errors and enhanced the reliability of the observed statistical associations.

### 2.7 Two-sample MR

A two-sample MR was used to detect potential causal associations between PM (PM2.5, PM2.5–10, and PM10) and neurodegenerative diseases (AD, ALS, DLB, PD, and MS). The inverse-variance weighting (IVW) method with different effect models served as the primary analysis method. A flowchart illustrating the methodology of this MR study is provided in Figure 1B.

To evaluate the robustness of the results, the following sensitivity analyses were performed: weighted median, simple mode, weighted mode, MR-Egger regression, and MR pleiotropy residual sum and outlier (MR-PRESSO) methods. It is noteworthy that these diverse methods yield valid results under distinct conditions. Specifically, the weighted median method provides a robust causal effect estimate even if up to 50% of the weights in the analysis originate from invalid IVs. The MR-Egger method is adept at detecting and correcting bias arising from directional pleiotropy, whereas the MR-PRESSO method can identify potential outlier IVs with horizontal pleiotropy. In cases where an outlier SNP was identified (*p* < 0.05) using MR-PRESSO, causal effects were recalculated by excluding the outliers. Additionally, funnel plots were generated to visually inspect the presence of pleiotropy, and Cochran’s Q test was used to evaluate heterogeneity among selected IVs. To explore the impact of individual SNPs on total estimates, a leave-one-out analysis was conducted by alternatively excluding each SNP. Furthermore, we used the MR Steiger test to ascertain the direction of causality; a “TRUE” result suggested causality in the expected direction.

### 2.8 MVMR analysis

To further ensure a comprehensive and reliable conclusion, we conducted an MVMR analysis to validate the causal relationships between PM2.5 exposure and AD that were identified in the two-sample MR study. In this analysis, we adjusted for the following potential risk factors: smoking, alcohol use, education, stroke, hearing loss, depression, and hypertension. Multivariable IVW, MR-Egger, and weighted median models were constructed in the MVMR. To establish causal relationships, statistical significance was determined as *p* < 0.05.

## 3 Results

### 3.1 Two-sample MR analysis

**Figure 2:**
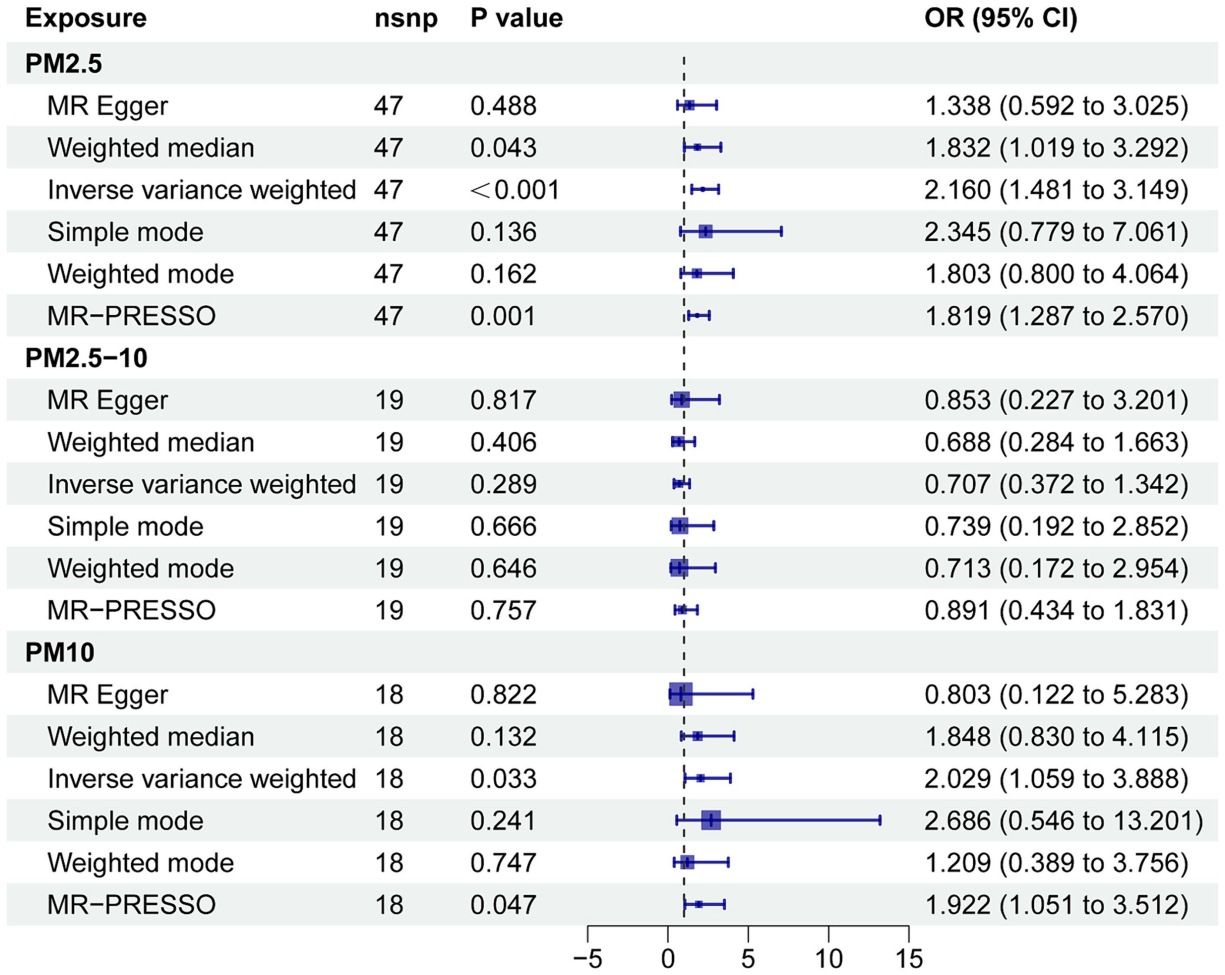
Forest plot for the effect of PM on AD. CI, confidence interval; MR-PRESSO, MR pleiotropy residual sum and outlier; OR, odds ratio.

**Figure 3:**
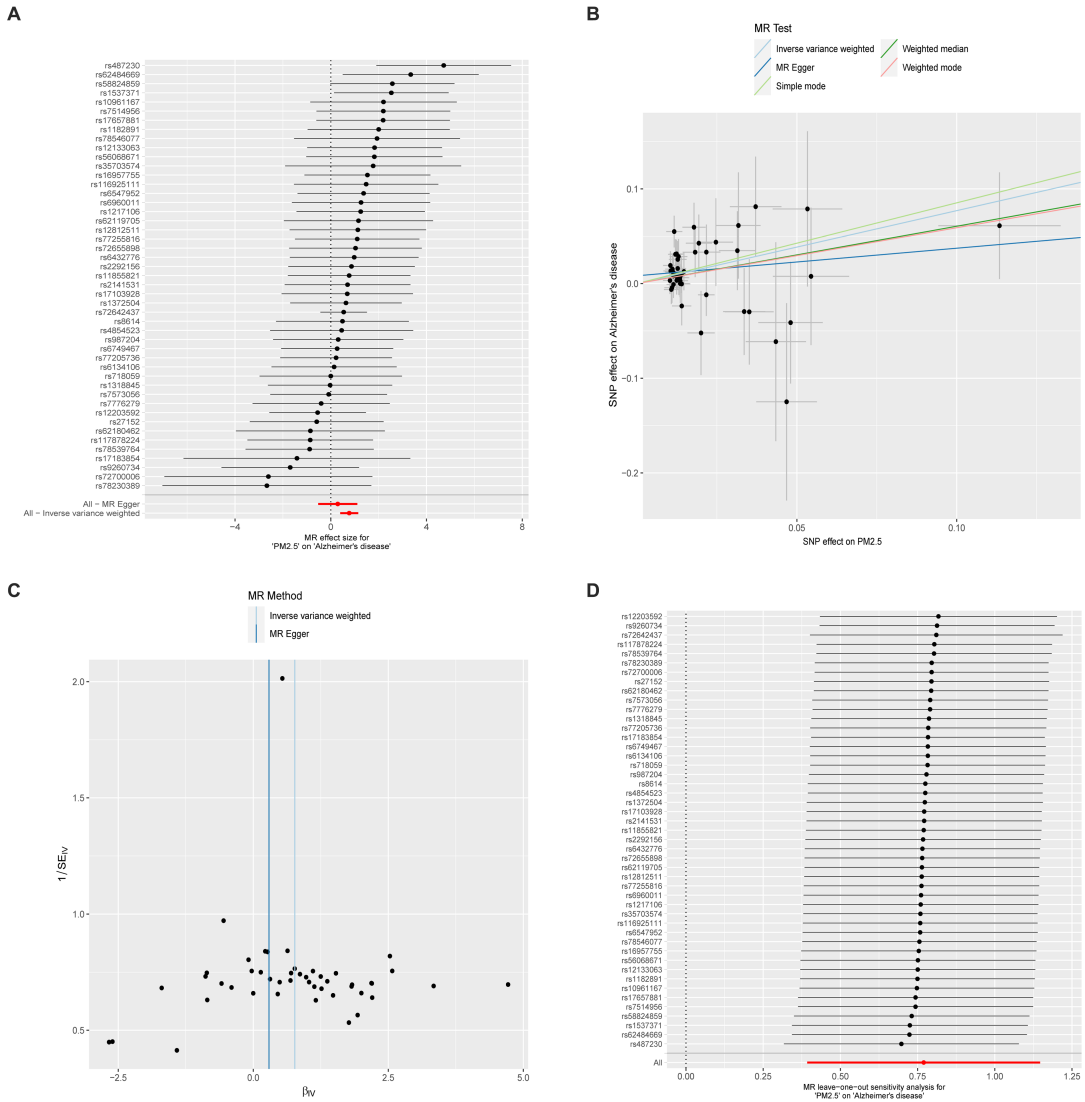
**(A)** Forest plot for the effect of each SNP on AD. **(B)** Scatter plot for the effect of PM on AD. **(C)** The overall heterogeneity test of the effect of PM on AD. **(D)** Leave-one-out to investigate whether the causal association was driven by a unique single SNP.

Supplementary Table S3 shows the results of the two-sample MR analysis to explore the causal effects of PM (PM2.5, PM2.5–10, and PM10) on neurodegenerative diseases (AD, ALS, DLB, PD, and MS). In the MR analysis based on the IVW method, a higher PM2.5 was related to an elevated risk of AD (odds ratio, 2.160; 95% confidence interval, 1.481 to 3.149; *p* < 0.001) (Figure 3A). The MR-PRESSO analysis reinforced the risk effect of PM2.5 on AD (odds ratio, 1.819; 95% confidence interval, 1.287 to 2.570; *p* = 0.001), thus corroborating the stability of the IVW results. In addition, consistency in directionality estimates was observed across the weighted median, simple mode, weighted mode, and MR-Egger regression methods (Figure 2). Figure 3B shows scatter plots of these analyses. The MR Steiger test further confirmed the direction of the causal relationship between PM2.5 and AD (*p* < 0.001; Supplementary Table S4). Additionally, PM10 exhibited a suggestive causal association with AD, whereas PM2.5–10 had no significant association with AD. All methods failed to detect any causal relationship between the three types of PM and ALS, DLB, MS, or PD.

### 3.2 Pleiotropy testing

To explore the potential impact of IVs on neurodegenerative diseases through alternative pathways, we conducted MR-Egger regression and MR-PRESSO global tests to assess the presence of pleiotropy. The MR-Egger regression tests revealed no evidence of directional pleiotropy for PM concerning neurodegenerative diseases, except for ALS. When investigating the causal relationship between PM2.5 and AD, the MR-PRESSO global test did not reveal any significant outliers. These findings suggest that the causal association between PM2.5 and AD is unaffected by pleiotropy. The detailed results are presented in Supplementary Tables S5, S6.

### 3.3 Heterogeneity testing

Supplementary Table S7 shows the results of Cochran’s Q tests for MR-Egger and IVW, which were performed to assess the heterogeneity of associations between exposures (PM2.5, PM2.5–10, PM10) and outcomes (AD, ALS, DLB, PD, and MS). Regardless of whether MR-Egger or IVW was used, there was no significant heterogeneity in the relationship between PM and AD, PD, or DLB. However, there was significant heterogeneity in the associations between PM2.5 and ALS and MS. In addition, clear heterogeneity was identified in genetic variants related to PM10 and MS. When we eliminate the outliers identified by MR-PRESSO test, the heterogeneity disappears. In fact, the presence of heterogeneity does not impact the non-significant association between PM2.5 and ALS or MS, nor between PM10 and MS. To further confirm the causal relationship between PM2.5 and AD, we constructed funnel plots; these showed no heterogeneous SNPs in our MR study (Figure 3C). Moreover, leave-one-out analysis revealed that the relationship between PM2.5 and AD was not influenced by a single SNP (Figure 3D).

### 3.4 MVMR analysis

**Figure 4:**
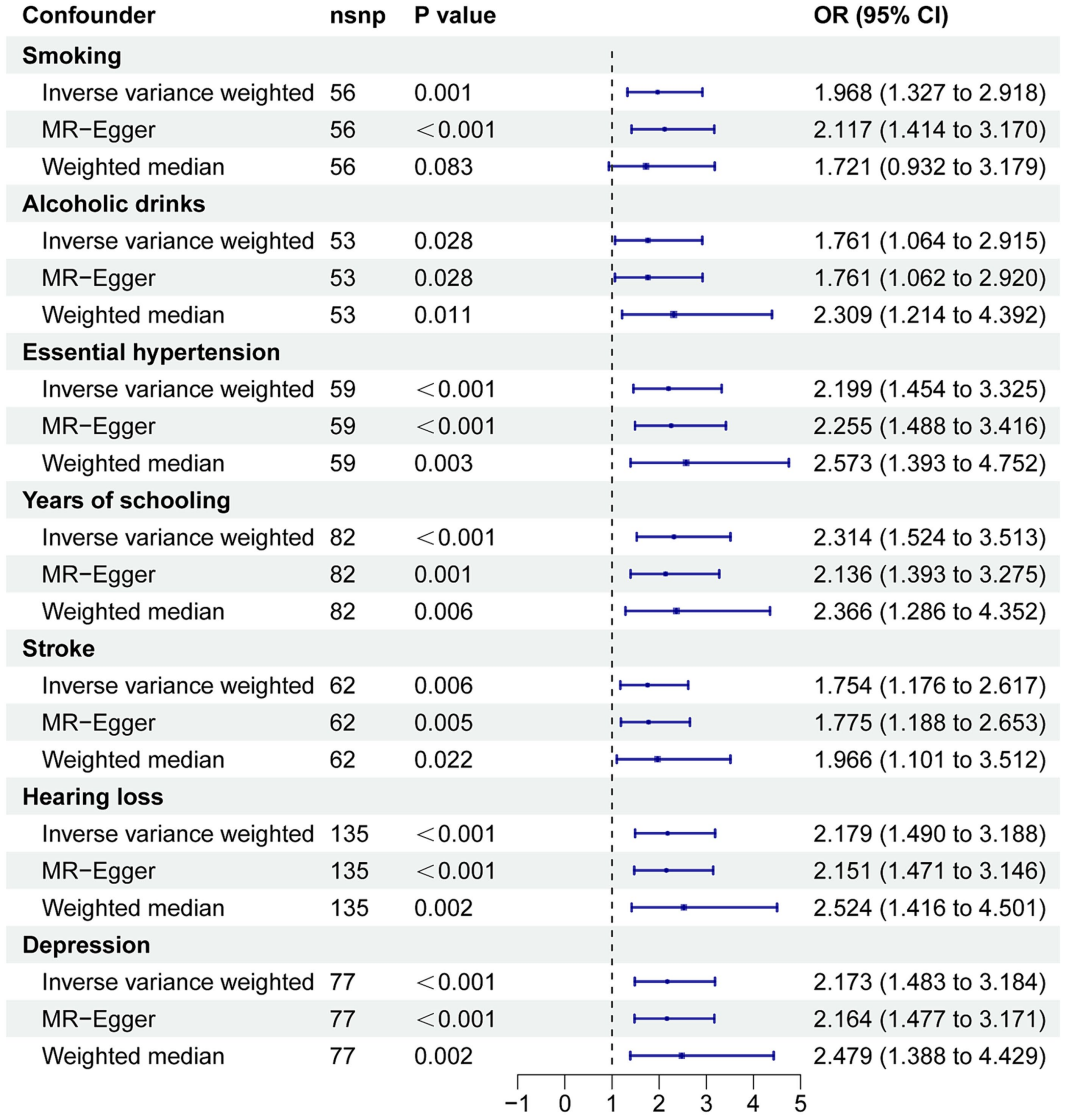
MVMR for the effect of PM2.5 on AD. CI, confidence interval; OR, odds ratio.

To further confirm the causal effect of PM2.5 on AD, we performed an MVMR analysis to adjust for smoking, alcohol use, education, stroke, hearing loss, depression, and hypertension. The results of this analysis revealed a causal relationship between PM2.5 on AD. This robust finding was further supported by sensitivity analyses, including MR-Egger and weighted median methods (as detailed in Figure 4).

## 4 Discussion

In the present study, we aimed to investigate the causal relationship between PM (PM2.5, PM2.5–10, and PM10) and five neurodegenerative diseases (AD, ALS, DLB, PD, and MS) within the European population. We took a two-sample MR and MVMR approach using publicly available summary statistics. Our analyses revealed a significant and consistent association between higher levels of PM2.5 and increased AD incidence. Importantly, this association remained robust even after adjustments for potential confounders such as smoking, alcohol use, education, stroke, hearing loss, depression, and hypertension. By contrast, PM had no significant associations with ALS, DLB, MS, or PD. Together, these findings contribute to improving our understanding of the complex interplay between different types of PM and neurodegenerative diseases. They also offer valuable insights for the development of targeted preventive strategies and therapeutic interventions specifically tailored for AD.

Air pollution is a major global health concern, and industrialization has contributed substantially to increased atmospheric PM levels, which are associated with adverse health effects. The World Health Organization declared the harmful health effects of PM in 2005 (22). PM is therefore a substantial public health concern, and more research is needed to reduce pollution levels and safeguard human health. Understanding the potential relationship between environmental factors and neurodegenerative diseases has become a focal point of recent research (7). A prevailing trend in the literature indicates a relatively strong association between PM exposure—notably, PM2.5—and AD pathology across diverse cohorts (23, 24). For example, a longitudinal cohort study encompassing 3.4 million cases of AD and related dementias identified a significant association between annual PM2.5 exposure and increased hazard ratios for first hospital admission for AD and related dementias (hazard ratio, 1.13; 95% confidence interval, 1.12 to 1.14) (25). Moreover, to evaluate the reliability of the association between prolonged PM2.5 exposure and diverse health outcomes, a comprehensive meta-analysis of 24 studies was conducted; the authors concluded that PM2.5 can increase the risk of stroke, lung cancer, and AD (26). PM2.5 exposure may also contribute to the accumulation of amyloid plaques in the brain and increase the risk of AD development (24). However, existing studies are marked by heterogeneity (such as divergent PM compositions, dosage levels, geographical regions of exposure, and durations) that introduce complexities into epidemiological studies. Considering the multifaceted nature of PM exposure parameters, a nuanced approach is needed for a comprehensive understanding of the health implications of PM.

When evaluating the health risks associated with exposure to environmental pollutants, delving into genetic variations can offer a more profound understanding of individual environmental health risks. For example, a genetics-centered study unveiled that women carrying the GPX4-rs376102 AC/CC genotype are particularly vulnerable to the impacts of air pollutants (27). As a result, genetic data can be harnessed to explore the causal link between PM and diseases. MR has emerged as an invaluable tool for exploring causal relationships between environmental exposures and complex diseases such as neurodegenerative disorders. By using genetic variants as IVs, MR mitigates issues arising from reverse causation and confounding biases, which commonly affect traditional epidemiological studies. In the present study, we used genetic variants associated with PM exposure as IVs to evaluate the potential causal impact of PM on neurodegenerative disease risk. The estimated causal effect, determined using the IVW method, suggests that higher PM2.5 exposure leads to an increased likelihood of developing AD. To ensure the robustness of our findings, we performed a comprehensive set of sensitivity analyses that considered potential pleiotropy and other biases that might influence the results. The consistent outcomes observed across these sensitivity analyses further enhance the validity and credibility of our observed association. Moreover, even after adjusting for potential confounding factors, the estimated causal effect remained significant. This finding underscores the likely direct and independent role of PM2.5 in the etiology of AD. However, a recent MR study found a non-significant association between PM2.5 and AD (28), differing from our results. The potential reason for the disparity could lie in the thresholds employed for selecting SNPs. They used *p* < 5 × 10^−8^, picking 8 SNPs, while we used *p* < 5 × 10^−6^, selecting 47 SNPs. Notably, *p* < 5 × 10^−6^ offers higher statistical power than *p* < 5 × 10^−8^ (99.9% vs. 38.9%) (Supplementary Table S8).^1^ Therefore, we believe that the threshold of 5 × 10^−6^ is suitable in our context.

Several potential pathways through which PM impacts the brain have been identified. Animal model studies have extensively explored the relationship between PM exposure and AD pathology. Exposure to PM elicits widespread transcriptional alterations in the brains of transgenic AD mice, thus expediting the progression of AD-associated pathology, including increased amyloid-β_42_ expression and tau phosphorylation (6). PM2.5 can also activate the nucleotide-binding domain and leucine-rich repeat protein 3 inflammasome, thus triggering the release of apoptosis-associated speck-like protein containing a caspase recruitment domain, which may accelerate amyloid-β aggregation (29). In addition, PM2.5 can traverse the blood–air barrier and subsequently increase dementia risk via detrimental neuroinflammation in the form of astrogliosis, microglial activation, and subsequent neuronal pathology (30). Similarly, long-term PM2.5 exposure in animal models intensifies neuroinflammation, characterized by reactive oxygen species production and pro-inflammatory cytokine release, resulting in neuronal damage (31). Evidence from clinical studies indicate that PM2.5 is associated with AD biomarkers, such as decreased brain-derived neurotrophic factor and elevated tau, in cerebrospinal fluid (32, 33). Moreover, recent studies have highlighted the impact of PM on gut microbiome homeostasis, which may give rise to neuroinflammation and neurodegenerative conditions (34). Another study has reported that PM exposure triggers systemic inflammation, neuroinflammation, and an increased metal load, thus collectively impairing glymphatic clearance efficiency. This compromised clearance mechanism may then accelerate AD progression (35). In summary, PM2.5 exposure likely exacerbates the aggregation and deposition of AD-related pathological hallmarks in the brain via mechanisms related to microglia/astrocyte activation, oxidative stress, immune activation, and neuroinflammation. Our MR results represent an important contribution to the expanding body of evidence linking PM2.5 exposure to AD. These findings underscore the importance of addressing air pollution as part of public health and AD prevention strategies.

To the best of our knowledge, the present study represents the first comprehensive MR-based exploration of the potential causal impacts of PM on neurodegenerative diseases. However, our study has some limitations. First, horizontal pleiotropy remains a significant challenge in MR analysis, and poses a potential source of bias. Despite our implementation of multiple measures to mitigate horizontal pleiotropy, we cannot completely disregard the impact of horizontal pleiotropy. This is attributed to the ambiguous biological functions of numerous genetic variations, which may lead to false positive outcomes. Second, our analysis primarily relied on data from individuals of European ancestry. Although we made this choice to mitigate bias caused by population stratification, it may restrict the generalizability of our findings to other ethnic groups. As the intake of air pollutants changes over time in tandem with shifts in people’s lifestyles and regional environmental protection measures, large-scale observational studies in real-world were needed to enhance the causal relationship between air pollution and AD in the future. Third, the assumption of a linear relationship in the MR analysis was based on summary-level data; this assumption limited our ability to explore the potential nonlinear roles of PM in neurodegenerative diseases. Moreover, although the MVMR approach considers potential confounders, the complex and multifaceted nature of AD pathogenesis poses challenges in capturing the entire causal pathway; this may lead to residual confounding or incomplete assessments.

## 5 Conclusion

The findings of the present study underscore the significant association between PM2.5 exposure and heightened AD risk. By contrast, PM did not increase the risk of ALS, DLB, MS, or PD. Our results highlight the critical importance of interventions to improve air quality as a pivotal public health strategy aimed at reducing AD risk.

## Data availability statement

The original contributions presented in the study are included in the article/Supplementary material, further inquiries can be directed to the corresponding authors.

## Author contributions

MD: Conceptualization, Supervision, Writing – original draft, Writing – review & editing. YL: Writing – original draft. LiZ: Writing – original draft. TL: Writing – original draft. ZL: Writing – original draft. JL: Writing – original draft. YF: Writing – original draft. YY: Writing – original draft. FL: Writing – original draft. FT: Writing – original draft. XW: Writing – original draft. YJ: Writing – original draft. HW: Writing – original draft. LeZ: Writing – original draft. HF: Writing – review & editing. GZ: Writing – review & editing.

## Footnotes

^1^ https://sb452.shinyapps.io/power/
